# P-1265. Tracking Resistance: Four years of Pseudomonas aeruginosa and its Battle with Antibiotics

**DOI:** 10.1093/ofid/ofaf695.1456

**Published:** 2026-01-11

**Authors:** David S Burgess, Katie B Olney, Donna R Burgess

**Affiliations:** University of Kentucky, Lexington, KY; University of Kentucky HealthCare, Lexington, KY; UK HealthCare, Lexington, KY

## Abstract

**Background:**

*Pseudomonas aeruginosa* is a difficult Gram-negative pathogen due to its intrinsic and acquired resistance. As beta-lactams remain central to treatment, local susceptibility is vital. This study evaluated susceptibility to first-line agents and newer beta-lactam agents over a 4-year period at our academic medical center.

**Methods:**

Non-duplicate *P. aeruginosa* isolates from January 2021 through December 2024 were analyzed. Susceptibility was determined by BD Phoenix™ or E-test and interpreted according to CLSI breakpoints. Antimicrobials analyzed included aztreonam (AZT), cefepime (CFP), piperacillin-tazobactam (PTZ), meropenem (MEM), ceftolozane-tazobactam (C/T), ceftazidime-avibactam (C/A), ciprofloxacin (CIP) and levofloxacin (LEV). Resistance phenotypes including MDR, XDR, DTR, and PDR were defined per CDC criteria. Culture source and hospital location were also recorded.

**Results:**

A total of 1,509 non-duplicate *P. aeruginosa* isolates were analyzed. The overall prevalence of resistance phenotypes was: MDR 18.2%, XDR 16.8%, DTR 5.0%, and PDR 0.9%. Respiratory specimens comprised the largest proportion of isolates (39.6%) compared to urine (23.7%), wound (12.9%), blood (12.6%), and other sources (9.1%).

Overall susceptibility rates were as follows: C/T 98.5%, C/A 97.8%, MEM 84.7%, CFP 83.9%, PTZ 79.6%, AZT 75.9%, CIP 72.3%, and LEV 69.9%. Of all isolates, 63.0% were susceptible to all first-line beta-lactams, 30.8% were non-susceptible to at least one, and 6.2% were non-susceptible to all. Among isolates non-susceptible to at least one beta-lactam, 41.7% were resistant to a single agent, 19.4% to two agents, 17.6% to three, and 21.3% to four. Respiratory isolates consistently demonstrated the highest rates of non-susceptibility across all agents tested.

**Conclusion:**

This analysis highlights the persistent challenge of *P. aeruginosa* resistance, particularly among respiratory isolates. The high in vitro activity of newer beta-lactams such as C/T and C/A supports their role in treatment algorithms for resistant infections. Continued local surveillance, antimicrobial stewardship, and individualized therapy remain essential for preserving the efficacy of beta-lactams in the management of *P. aeruginosa*.

**Disclosures:**

All Authors: No reported disclosures

